# Gastrointestinal Metastases From Lobular Breast Carcinoma: A Literature Review

**DOI:** 10.7759/cureus.65852

**Published:** 2024-07-31

**Authors:** Zacharoula Kioleoglou, Eleni Georgaki, Nektarios Koufopoulos, Osman Kostek, Nikolaos Volakakis, Areti Dimitriadou, Stefania Kokkali

**Affiliations:** 1 Department of Surgery, Metaxa Cancer Hospital, Piraeus, CYP; 2 Second Department of Medicine, Medical School, Hippocratio General Hospital of Athens, National and Kapodistrian University of Athens, Athens, GRC; 3 Second Department of Pathology, Medical School, Attikon University Hospital, National and Kapodistrian University of Athens, Athens, GRC; 4 Department of Internal Medicine, Division of Medical Oncology, Sultan 1 Murad State Hospital, Edirne, TUR; 5 Oncology Unit, General Hospital of Athens Hippocrates, Athens, GRC

**Keywords:** metastatic breast cancer, invasive lobular carcinoma, gastrointestinal metastases, gastric metastasis, duodenal metastasis, colonic metastasis

## Abstract

Invasive lobular carcinoma (ILC) represents a rare subtype of breast carcinoma, originating from the lobule. Unlike ductal carcinoma, ILC does not express E-cadherin and thus can metastasize to uncommon sites. We aimed to investigate the clinicopathological characteristics of the rare subgroup of ILC patients with gastrointestinal (GI) metastases. A PubMed search was undertaken using the terms “Lobular Breast Carcinoma” AND “Gastrointestinal Metastasis.”

We identified 169 cases, with metachronous GI metastatic disease being approximately twice as common as synchronous GI metastases. The median age at initial diagnosis was 56.7 years (24-88). The majority of patients were hormonal receptor-positive and only a small minority was HER2-positive. The appearance of a gastrointestinal lesion was often the mode of revelation of ILC. Differential diagnosis from primary gastrointestinal cancer is sometimes challenging, especially in the case of signet-ring cell carcinoma. The median time from breast cancer diagnosis to GI metastases was 6.5 years (0-33). Most common metastatic sites include the stomach, colon, and rectum, in order of decreasing frequency, whereas metastases were found in every part of the digestive tract. In conclusion, metastases of ILC can arise in the gastrointestinal tract and they should be managed similarly to metastatic breast cancer.

## Introduction and background

Invasive lobular carcinoma (ILC) of the breast is the second most common type of invasive breast carcinoma which accounts for approximately 10% of all breast cancers [1], with an increasing incidence, especially among postmenopausal females [2]. Histopathologic pattern of ILC differs from invasive ductal carcinoma (IDC) and is characterized by small round cells infiltrating the stroma of the breast as individual rows (single or Indian file) [3]. 

This type of infiltration generally does not disrupt anatomical structures and does not induce any substantial conjunctive tissue response. Therefore, ILC often does not form a distinct mass in the breast and diagnosis can be challenging by palpation or mammography [4]. It has a tendency for a multifocal, multicentric, and bilateral distribution [5,6]. There are several histopathological variants, including classic, solid, alveolar, tubuloglobular, pleomorphic, and mixed. Notably, the pleomorphic variant may show apocrine or histiocytoid differentiation and may be composed of signet ring cells [7].

ILC has almost invariably positive hormone receptors and HER2 positivity is very rare, generally limited to the pleomorphic variant. In addition, E-cadherin is usually absent or reduced in ILC but 10-16% of ILC may express E-cadherin with unknown prognostic significance [8]. It is suggested that ILC has a higher rate of distant metastases, in comparison to IDC, probably due to its infiltrative nature [9,10]. It is known that loss of E-cadherin, the molecule of intercellular adhesion, may facilitate the metastatic process. Similarly to the breast, metastatic ILC tends to infiltrate the affected organs in a diffuse pattern, instead of forming a well-defined tumoral nodule. 

Interestingly, ILC patterns of metastasis differ significantly from IDC. Distinctive metastatic sites of ILC include intraabdominal serosal surfaces, gastrointestinal tract, genitourinary system organs, and leptomeninges, whereas pulmonary metastases are less frequent, for unknown reasons, while the most common sites of metastasis of IDC involve the bone, liver, and lungs [4,9,11,12]. Gastrointestinal metastasis (GI) of ILC constitutes a clinical challenge for physicians since primary gastrointestinal malignancy is considered a differential diagnosis.

Metastatic breast cancer is treated with systemic therapy, including chemotherapy, endocrine therapy, and targeted therapy (anti-HER2, PARP inhibitors, etc.). Given that most patients with GI metastases from ILC are positive for hormone receptors, endocrine therapy is the recommended first-line treatment, providing favorable outcomes in terms of survival and response rate. There is no adequate data describing the features of this rare clinical entity in the literature. The goal of this review is to analyze the clinicopathological characteristics of ILC patients with GI metastases.

This article was previously presented as a meeting abstract at the 20th World Congress of SIS on December 6-7, 2018.

## Review

Methods

A protocol for systemic review of published articles was used to evaluate the cases of gastrointestinal metastases from ILC. An online search of the PubMed database using the terms "Lobular Breast Carcinoma" AND "Gastrointestinal Metastasis" was conducted on 31 August 2023. Among the retrieved papers selected for the study, we included only the ones referring to metastases in the gastrointestinal tract (esophagus, stomach, duodenum, small bowel, colon, and rectum) and excluded articles describing hepatic, vesicular, pancreatic, and splenic metastases. We included cases of pure lobular carcinoma of the breast, as well as mixed cases that included a lobular component. We identified mainly case reports and some case series. Other eligibility criteria were the availability of sufficient clinicopathological data and articles written in the English language. Posters and animal studies were excluded.

We collected histopathological information on both the primary breast tumor and gastrointestinal metastasis, including biomarkers estrogen receptor (ER), progesterone receptor (PR), and human epidermal growth factor receptor 2 (HER2). ER and PR were classified as positive or negative, according to the conclusion of each laboratory. In the case of HER2 score of 2+, we searched for fluorescence in situ hybridization (FISH) or chromogenic in situ hybridization (CISH) results. Clinical information was also registered concerning the stage at first diagnosis and the initial management of breast tumors in case of metachronous metastases.

The exact locations of both gastrointestinal and other metastases were registered. We analyzed separately the symptoms and signs associated with gastrointestinal metastases, as well as their treatment (local or other) and prognosis.

This study represents a narrative synthesis of the available literature. Tables are used to summarize the characteristics and findings of the included studies. For this qualitative study, we used simple descriptive statistics methods. Only cases with available data were included in the statistical analysis and different percentages refer to this population. For time-to-event analysis, the Kaplan-Meier method was used. Statistical analyses were computed using SPSS version 28 (Armonk, NY: IBM Corp.).

Results

A total of 169 cases were incorporated into this analysis, including 85 case reports and six case series (Table 1) [13-98]. Three out of the six case series, including 64 patients in total, represent pooled analyses of cases and do not contain any individual clinical and histological information [93,96,97].

**Table 1:** Characteristics and management of primary breast tumors. NM: not mentioned; POS: positive; NEG: negative; NR: not realized; NA: not applicable; FAC: 5-fluorouracil/adriamycin/cyclophosphamide; AC: adriamycin/cyclophosphamide; ECMF: epirubicin/cyclophosphamide/methotrexate/5-fluorouracil; EC: epirubicin/cyclophosphamide; R: right; L: left; axil: axillary; SLND: sentinel lymph node dissection; cerv: cervical; TAM: tamoxifen; AI: aromatase inhibitor; adj: adjuvant; IHC: immunohistochemistry; TC: taxotere/cyclophosphamide; Bilat: bilateral; FEC: 5-fluorouracil, epirubicin, and cyclophosphamide

Case no.	Studies	ER (breast)	PR (breast)	HER2 (breast)	Stage at diagnosis	Chemo (adj)	RT (adj)	Breast surgery	HT (adj)
1	Pera et al. [13]	POS	POS	NM	II	No	Yes	L mastectomy, axil LND	NM
2	Cervi et al. [14]	NM	NM	NM	I	No	No	Mastectomy	No
3	Jones et al. [15]	POS	POS	NM	II	No	Yes	R lumpectomy, axil LND	TAM, AI, ovariectomy
4	Jones et al. [15]	POS	POS	NM	III	Yes	Yes	L mastectomy, axil LND	TAM
5	Nihon-Yanagi et al. [16]	POS	POS	NEG	II	Yes	No	L mastectomy, axil LND	TAM
6	Sato et al. [17]	NEG	NEG	NEG	IV	NA	NA	No	NA
7	Puglisi et al. [18]	POS	POS	POS	IV	No	Yes	R lumpectomy, axil, and cerv LND	TAM
8	Vennapusa et al. [19]	NM	NM	NM	IV	NA	NA	No	NA
9	López Deogracias et al. [20]	POS	POS	NM	II	Yes	Yes	L mastectomy, axil LND	No
10	Amin et al. [21]	NM	NM	NM	Primary	No	No	R mastectomy, SLND	TAM
11	Okido et al. [22]	NEG	POS	2+ IHC	III	Yes	Yes	R mastectomy, axil LND	TAM
12	Koike et al. [23]	POS	POS	NEG	III	AC, paclitaxel	No	Mastectomy, axil LND	AI
13	Koike et al. [23]	POS	POS	NEG	IV	NA	NA	No	NA
14	Saied et al. [24]	NM	NM	NM	III	Yes	Yes	R lumpectomy, axill LND	TAM, letrozole
15	Zhao et al. [25]	POS	NM	NEG	III	Yes	Yes	L mastectomy, axil LND	Letrozole
16	Critchley et al. [26]	POS	NM	NM	III	ECMF	Yes	Mastectomy, axil LND	TAM, letrozole
17	Saranovic et al. [27]	POS	POS	NEG	IV	NA	NA	No	NA
18	Arrangoiz et al. [28]	POS	NEG	NEG	IV	NA	NA	No	NA
19	Carcoforo et al. [29]	POS	POS	NEG	IV	NA	NA	No	NA
20	Zuhair and Maron [30]	NM	NM	NM	IV	NA	NA	Bilat mastectomy	NA
21	Eren et al. [31]	POS	POS	NM	IV	Yes	NA	No	Yes
22	Kayılıoğlu et al. [32]	NR	NR	NR	IV	NA	NA	No	NA
23	Jones et al. [33]	NR	NR	NR	IV	NA	NA	No	NA
24	Molina-Barea et al. [34]	POS	NEG	NEG	Primary	FAC	Yes	L mastectomy, axil LND	No
25	Geredeli et al. [35]	NEG	NEG	NEG	IV	NA	NA	No	NA
26	Dória et al. [36]	POS	POS	NEG	IV	NA	NA	No	NA
27	Buka et al. [37]	POS	POS	NEG	IV	Docetaxel, EC	Yes	No	TAM
28	Balakrishnan et al. [38]	POS	POS	NEG	III	TC	Yes	Bilat mastectomy	AI
29	Villa Guzmán et al. [39]	POS	POS	NEG	III	AC, docetaxel	Yes	Lumpectomy, axil LND	Exemestane
30	Lau et al. [40]	NM	NM	NM	I	No	No	R mastectomy	TAM
31	Khokhlova et al. [41]	NEG	NEG	NEG	I	No	No	R mastectomy, SLND	No
32	Cherian et al. [42]	POS	NM	NM	III	No	Yes	R mastectomy, axil LND	Anastrozole
33	Horimoto et al. [43]	POS	POS	NEG	III	Docetaxel	No	L mastectomy, axil LND	TAM, AI
34	Xu et al. [44]	POS	POS	NEG	IV	NA	NA	No	NA
35	Koufopoulos et al. [45]	POS	POS	NR	NM	No	No	No	No
36	Invento et al. [46]	ΝΜ	ΝΜ	ΝΜ	ΝΜ	ΝΜ	ΝΜ	R mastectomy	NM
37	Asmar et al. [47]	POS	POS	NEG	NM	Yes	Yes	L mastectomy	Yes
38	Ogawa et al. [48]	POS	NEG	NEG	IV	NA	NM	R mastectomy	NA
39	Falco et al. [49]	POS	POS	NEG	III	EC, docetaxel	NM	L mastectomy, axil LND	TAM, letrozole
40	Ruymbeke et al. [50]	POS	POS	NEG	IV	NA	NA	Bilat mastectomy	TAM
41	Woo et al. [51]	POS	POS	NEG	IV	EC, docetaxel	Yes	Bilat mastectomy, axil LND	Letrozole
42	Figueiredo at al. [52]	POS	POS	NM	IV	NA	NA	No	NA
43	Mosiun et al. [53]	NEG	NEG	Equivocal	III	FEC, docetaxel	Yes	R mastectomy, axil LND	No
44	Sharbatji et al. [54]	POS	POS	NEG	Primary	Yes	No	Mastectomy	Yes
45	Numan et al. [55]	POS	POS	NEG	Primary	No	No	Mastectomy	Yes
46	Blachman-Braun et al. [56]	NM	NM	NM	Primary	Yes	NM	Mastectomy	NM
47	Gangireddy et al. [57]	POS	NM	NEG	Primary	Yes	Yes	Lumpectomy	Yes
48	Noor et al. [58]	POS	POS	NEG	NM	Yes	No	Bilat mastectomy	Yes
49	Sarfraz et al. [59]	POS	POS	NEG	NM	No	No	Bilat mastectomy	Yes
50	Meekel et al. [60]	POS	NEG	NEG	IIB	Yes	Yes	R lumpectomy	Yes
51	Kaneko et al. [61]	POS	NM	NEG	IIIB	Yes	Yes	Mastectomy, axil LND	Yes
52	Kobayashi et al. [62]	NM	NM	NM	IIA	No	No	R lumpectomy	No
53	Fu et al. [63]	POS	POS	NEG	IA	Yes	No	L mastectomy, axil LND	Yes
54	Kutasovic et al. [64]	POS	POS	NEG	NM	No	Yes	Lumpectomy, axil LND	Yes
55	Fontes et al. [65]	POS	NEG	NEG	IV	NΑ	NΑ	No	NA
56	Alfian Sulai et al. [66]	NM	NM	NM	IV	Yes	Yes	R lumpectomy, axil LND	Yes
57	Abdallah et al. [67]	POS	POS	NEG	IV	Yes	NΑ	No	Yes
58	Abdallah et al. [67]	NR	NR	NR	IV	ΝΑ	ΝΑ	No	NA
59	Maharajh et al. [68]	NM	NM	NM	IIIA	Yes	Yes	L mastectomy, axil LND	Yes
60	Ali et al. [69]	POS	POS	NEG	IV	NΑ	NΑ	No	NA
61	Almahmeed et al. [70]	POS	POS	NEG	IV	NΑ	NΑ	No	NA
62	Zhang et al. [71]	POS	POS	POS (1+)	IV	NΑ	NΑ	No	NA
63	Algethami et al. [72]	POS	POS	NEG	NM	No	Yes	Bilat mastectomy, axil LND	Yes
64	Mazza et al. [73]	NEG	NEG	NEG	IV	NA	NA	No	NA
65	Zengel et al. [74]	POS	NEG	POS (1+)	IIB	Yes	Yes	R mastectomy, axil LND	No
66	Zengel et al. [74]	POS	POS	NEG	IV	NA	NA	No	NA
67	Imai et al. [75]	POS	NM	NM	IV	NA	NA	No	NA
68	Kimchy et al. [76]	NEG	NEG	NEG	IV	NA	NA	No	NA
69	Katuwal et al. [77]	POS	NEG	NEG	NM	No	No	No	Yes
70	Sun et al. [78]	POS	NEG	NEG	IV	NA	NA	No	NA
71	Martino et al. [79]	POS	NEG	POS (2+)	IIB	Yes	No	Bilat mastectomy, axil LND	Yes
72	Shin et al. [80]	POS	POS	NEG	IIIB	Yes	Yes	R mastectomy	Yes
73	Anguiano-Albarran et al. [81]	POS	NEG	NEG	NM	No	Yes	L mastectomy	No
74	Ito et al. [82]	POS	POS	NEG	IV	NA	NA	No	NA
75	Ito et al. [82]	POS	POS	NEG	NM	Yes	Yes	L mastectomy	Yes
76	Yoshida et al. [83]	NM	NM	NM	NM	Yes	No	Bilat mastectomy, axil LND	Yes
77	Li et al. [84]	POS	POS	NEG	IIB	Yes	Yes	R mastectomy, SLND	Yes
78	Li et al. [85]	NM	NM	NM	II	Yes	Yes	R mastectomy, axil LND	Yes
79	Li et al. [86]	POS	POS	NEG	IV	NA	NA	Bilat mastectomy	NA
80	Barbieri et al. [87]	POS	POS	NEG	IIIB	Yes	Yes	L lumpectomy, axil LND	Yes
81	Kachi et al. [88]	POS	POS	NEG	Primary	Yes	Yes	Mastectomy	Yes
82	Namikawa et al. [89]	POS	NM	NEG	IV	NA	NA	No	NA
83	Yanagisawa et al. [90]	POS	NM	NEG	II	No	Yes	R lumpectomy, SLND	Yes
84	Skafida et al. [91]	POS	POS	NEG	IV (chest wall skin)	Yes	Yes	Mastectomy	Yes
85	Bashir Hamidu et al. [92]	POS	POS	NEG	NM	Yes	Yes	L mastectomy, axil LND	Yes
86-112	McLemore et al. [93]	NM	NM	NM	Primary	NM	NM	NM	NM
113-119	Ayantunde et al. [94]	NM	NM	NM	Primary	NM	NM	Yes	NM
120-125	Pectasides et al. [95]	NM	NM	NM	NM	NM	NM	NM	NM
126-132	Mathew et al. [96]	NM	NM	NM	NM	NM	NM	NM	NM
133-162	Montagna et al. [97]	NM	NM	NM	NM	NM	NM	NM	NM
163	Hong et al. [98]	POS	NEG	NEG	IV	NA	NA	No	NA
164	Hong et al. [98]	POS	POS	NEG	NM	Yes	Yes	Mastectomy	Yes
165	Hong et al. [98]	POS	POS	NEG	NM	Yes	No	No	Yes
166	Hong et al. [98]	POS	POS	NEG	NM	Yes	Yes	Mastectomy	Yes
167	Hong et al. [98]	POS	POS	NEG	NM	Yes	Yes	Mastectomy	Yes
168	Hong et al. [98]	POS	POS	NEG	NM	Yes	Yes	Mastectomy	Yes
169	Hong et al. [98]	POS	POS	NEG	NM	Yes	Yes	Mastectomy	Yes

Age at initial diagnosis (of either primary breast cancer or metastatic disease) varied between 24 and 88 years, with a median value of 56.7. All patients were females. Among cases with known ER status (n=76), 90.8% (n=69) of mammary tumors were positive. Among cases with known PR status (n=68), 75% (n=51) of mammary tumors were positive. Among cases with known HER2 status (n=65), 93.8% (n=61) were negative, 4.6% (n=3) positive, and 1.5% (n=1) equivocal (Table 1). There are also five patients with triple-negative breast carcinoma [17,35,41,73,76]. All breast tumors were lobular carcinomas, apart from eight cases with mixed ductal and lobular histology [22,43,85,93,97].

For 109 patients the initial stage of diagnosis was available. Among them, in almost one female out of three (33%) initial diagnosis was made at stage IV. In the majority of these cases, gastrointestinal metastasis was the mode of revelation of breast carcinoma. For the rest of the patients, primary breast cancer preceded the diagnosis of gastrointestinal metastases. For less than half of the patients of the latter group exact stage is available, with the majority diagnosed with stage III disease (n=16).

All primary breast tumors were treated with surgery and eight additional metastatic patients also underwent breast surgery. Among the 99 cases with available information on surgery, 50 (50.5%) underwent mastectomy, 10 (10.1%) lumpectomy, seven (7.1%) breast surgery with no further information, and 31 (31.3%) did not receive any surgery due to stage IV disease. In most patients, (sentinel) lymph node dissection was also performed. Of the 73 patients with stage I-III disease at diagnosis, 29 (39.7%) received adjuvant chemotherapy, mainly taxanes and anthracyclines, 10 (13.7%) did not receive chemotherapy, and the information was not available for the remaining patients. Forty-one patients received adjuvant radiation therapy (RT) to the breast, 25 of them had early breast cancer, five had stage IV disease, and in 11 cases the stage at the time of RT was not mentioned. Adjuvant hormonal therapy was reported in 34 patients, six of whom received aromatase inhibitors (AI), eight tamoxifen, five with tamoxifen switched to AI, and for 15 patients the type of hormonal therapy is not known.

Time from initial diagnosis to metastases varies between 0 and 33 years, with a median value of five years (95% CI: 4.23-5.77). In the majority of cases, histological diagnosis of metastasis was similar to breast tumor, except for six diagnoses of poorly differentiated carcinoma [15,16,20,26,33] and one of epithelioid neoplasm [19]. Similarly, receptor status does not vary significantly between primary breast carcinoma and GI metastasis, with 86.3% ER-positive (n=82), 55.4% PR-positive (n=36), and 81.6% (n=40) HER2-negative metastatic tumors, among patients with available information on receptors status (Table 2). Six additional GI metastases were HER2-positive and two HER2-low (Table 2). GI metastases were found in every part of the digestive tract, including the esophagus/cardia (4.7%), stomach/duodenum/ampulla of Vater (48.1%), jejunum/small intestine (4.7%), colon (17.9%), rectum/anus (10.4%), or in more than one location of the GI tract (14.2%).

**Table 2:** Characteristics of gastrointestinal metastases. GI: gastrointestinal; DFS: disease-free survival; POS: positive; NEG: negative; NM: not mentioned; NR: not realized

Case no.	Studies	DFS (years)	ER (GI)	PR (GI)	HER2 (GI)
1	Pera et al. [13]	7	POS	POS	NM
2	Cervi et al. [14]	8	POS	POS	NM
3	Jones et al. [15]	3	POS	POS	NM
4	Jones et al. [15]	14	POS	POS	NEG
5	Nihon-Yanagi et al. [16]	1	NEG	NEG	NM
6	Sato et al. [17]	Synchronous	NEG	NEG	NEG
7	Puglisi et al. [18]	4	POS	POS	NEG
8	Vennapusa et al. [19]	Synchronous	POS	NM	NM
9	López Deogracias et al. [20]	15	NEG	NEG	NM
10	Amin et al. [21]	17	POS	POS	NEG
11	Okido et al. [22]	5	NEG	NEG	POS
12	Koike et al. [23]	Synchronous	POS	POS	POS
13	Koike et al. [23]	Synchronous	POS	POS	NM
14	Saied et al. [24]	12	POS	POS	NEG
15	Zhao et al. [25]	3	POS	POS	NM
16	Critchley et al. [26]	8	POS	POS	NEG
17	Saranovic et al. [27]	6	POS	NM	NM
18	Arrangoiz et al. [28]	Synchronous	POS	POS	NM
19	Carcoforo et al. [29]	Synchronous	NM	NM	NM
20	Zuhair and Maron [30]	Synchronous	POS	NEG	NEG
21	Eren et al. [31]	5	POS	NM	NM
22	Kayılıoğlu et al. [32]	Synchronous	POS	POS	NM
23	Jones et al. [33]	Synchronous	POS	NEG	NEG
24	Molina-Barea et al. [34]	5	POS	NEG	NM
25	Geredeli et al. [35]	3	NEG	NEG	NEG
26	Dória et al. [36]	Synchronous	POS	POS	NM
27	Buka et al. [37]	Synchronous	POS	POS	NM
28	Balakrishnan et al. [38]	2.5	NM	NM	NM
29	Villa Guzmán et al. [39]	4	POS	POS	NEG
30	Lau et al. [40]	11	POS	POS	NM
31	Khokhlova et al. [41]	8	NEG	NEG	NEG
32	Cherian et al. [42]	10	POS	NM	NM
33	Horimoto et al. [43]	5	POS	NEG	NEG
34	Xu et al. [44]	Synchronous	POS	POS	NM
35	Koufopoulos et al. [45]	Synchronous	POS	POS	NR
36	Invento et al. [46]	16	NM	NM	NM
37	Asmar et al. [47]	20	POS	NEG	NEG
38	Ogawa et al. [48]	5	POS	NM	NM
39	Falco et al. [49]	14	POS	POS	NEG
40	Ruymbeke et al. [50]	4	POS	POS	NEG
41	Woo et al. [51]	1	POS	NEG	NM
42	Martins Figueiredo et al. [52]	Synchronous	NM	NM	NM
43	Mosiun et al. [53]	16	POS	POS	POS focally
44	Sharbatji et al. [54]	10	NEG	NM	NEG
45	Numan et al. [55]	3	NM	NM	NM
46	Blachman-Braun et al. [56]	15	POS	NEG	NEG
47	Gangireddy et al. [57]	24	POS	POS	NEG
48	Noor et al. [58]	27	POS	POS	NEG
49	Sarfraz et al. [59]	5	POS	POS	NEG
50	Meekel et al. [60]	0	NM	NM	NM
51	Kaneko et al. [61]	7	POS	NM	NM
52	Kobayashi et al. [62]	23	POS	NM	NM
53	Fu et al. [63]	9	POS	NM	NM
54	Kutasovic et al. [64]	2	NEG	NEG	NEG
55	Fontes et al. [65]	NM	NEG	NM	NM
56	Alfian Sulai et al. [66]	NM	NM	NM	NM
57	Abdallah et al. [67]	NM	POS	NM	NM
58	Abdallah et al. [67]	0	POS	NM	NM
59	Maharajh et al. [68]	2	POS	NEG	NEG
60	Ali et al. [69]	NM	POS	POS	POS
61	Almahmeed et al. [70]	NM	POS	POS	NEG
62	Zhang et al. [71]	NM	POS	POS	NM
63	Algethami et al. [72]	3.5	POS	NM	POS
64	Mazza et al. [73]	NM	NEG	NEG	NEG
65	Zengel et al. [74]	9	POS	NEG	LOW (1+)
66	Zengel et al. [74]	NM	POS	POS	NEG
67	Imai et al. [75]	NM	POS	NM	NM
68	Kimchy et al. [76]	NM	NEG	NEG	NEG
69	Katuwal et al. [77]	14	POS	NEG	NEG
70	Sun et al. [78]	NM	POS	NEG	NM
71	Martino et al. [79]	5	NM	NM	NM
72	Shin et al. [80]	3	POS	NEG	NEG
73	Anguiano-Albarran et al. [81]	2	POS	NEG	NEG
74	Ito et al. [82]	NM	NM	NM	NM
75	Ito et al. [82]	10	NM	NM	NM
76	Yoshida et al. [83]	6	POS	NM	NM
77	Li et al. [84]	1.5	POS	POS	NEG
78	Li et al. [85]	3	NM	NM	NM
79	Li et al. [86]	0	POS	POS	NR
80	Barbieri et al. [87]	5	POS	NEG	NEG
81	Kachi et al. [88]	5	POS	POS	NEG
82	Namikawa et al. [89]	0	POS	NM	NEG
83	Yanagisawa et al. [90]	NM	POS	POS	NEG
84	Skafida et al. [91]	2.8	POS	POS	NEG
85	Hamidu et al. [92]	5	POS	NEG	POS
86-112	McLemore et al. [93]	7	NM	NM	NM
113	Ayantunde et al. [94]	6.5	POS	NM	NM
114	Ayantunde et al. [94]	20	POS	NM	NM
115	Ayantunde et al. [94]	33	POS	NM	NM
116	Ayantunde et al. [94]	3	POS	NM	NM
117	Ayantunde et al. [94]	5.5	POS	NM	NM
118	Ayantunde et al. [94]	5	POS	NM	NM
119	Ayantunde et al. [94]	10.5	POS	NM	NM
120	Pectasides et al. [95]	3	POS	NM	NM
121	Pectasides et al. [95]	5	POS	NM	NM
122	Pectasides et al. [95]	Synchronous	POS	NM	NM
123	Pectasides et al. [95]	4	POS	NM	NM
124	Pectasides et al. [95]	7	POS	NM	NM
125	Pectasides et al. [95]	4	POS	NM	NM
126-132	Mathew et al. [96]	NM	NM	NM	NM
133-162	Montagna et al. [97]	NM	NM	NM	NM
163	Hong et al. [98]	Synchronous	POS	NM	NEG
164	Hong et al. [98]	13	POS	NEG	NEG
165	Hong et al. [98]	8	NEG	NEG	NM
166	Hong et al. [98]	7	POS	POS	NM
167	Hong et al. [98]	4	POS	POS	NEG
168	Hong et al. [98]	3.5	POS	NEG	NEG
169	Hong et al. [98]	10.4	NEG	NEG	NEG

Symptoms associated with GI metastases did not differ from the ones associated with primary tumors of the GI tract. Therefore, diagnosis of metastatic disease is challenging and purely histological. The most common symptoms were general digestive disorders (46.3%), abdominal pain (23.8%), and bowel obstruction (17.5%). Depending on the exact location of metastasis, other symptoms were also reported, including changes in bowel habits, nausea and vomiting, weight loss, anorexia, dysphagia, anemia, rectorrhagia, jaundice, fatigue, and abdominal mass. In the majority of cases, there was a combination of symptoms.

GI metastases appeared simultaneously with other metastases in approximately two-thirds of patients with available data (n=105), whereas 39 patients (37.1%) presented no other metastases. Extra-digestive metastatic locations included the bones (38.1%), peritoneum (19%), lymph nodes (10.5%), ovaries (8.6%), liver (7.6%), brain (6.7%), or other. Surprisingly, the lungs/pleura were only affected in 3.8% of the patients. Nineteen percent of the patients presented with multiple locations involved (Table 3).

**Table 3:** Management of gastrointestinal metastases and outcome. Systemic treatment refers to chemotherapy or hormonal treatment given after gastrointestinal metastasis diagnosis. When there are no further details on the type of hormonal treatment or chemotherapy administered, "chemo" or "hormonal" is reported in the column "systemic treatment," respectively. Transverse and sigmoid refer to the colon. Survival after GI metastasis is expressed in years GI: gastrointestinal; hormonal: no further details; Chemo: chemotherapy; RT: radiation therapy; R: right; L: left; LN: lymph node; LND: lymph node dissection; BM: bone marrow; AI: aromatase inhibitor; FAC: fluorouracil-adriamycin-cyclophosphamide; CMF: cyclophosphamide-methotrexate-fluorouracil; TAM: tamoxifen; BEV: bevacizumab; xeliri: xeloda (capecitabine) irinotecan; AC: adriamycin-cyclophosphamide; BSC: best supportive care; EC: epirubicin-cyclophosphamide

Case no.	Studies	GI metastasis	Other metastatic sites	RT to metastasis	Systemic treatment	GI intervention	Survival after GI met (years)
1	Pera et al. [13]	Stomach	No	No	Hormonal	Selective gastrectomy	NM
2	Cervi et al. [14]	Rectum	No	No	NM	Abdominoperineal resection	NM
3	Jones et al. [15]	Stomach	Bone	No	Hormonal	D2 gastrectomy	NM
4	Jones et al. [15]	Cardia	Bone, plevra, brain	Cerebral	Chemo, AI	No	NM
5	Nihon-Yanagi et al. [16]	Duodenum	No	No	Capecitabine, TAM	Whipple	<1
6	Sato et al. [17]	Duodenum	Bone, LN, peritoneum	No	Paclitaxel, anthracyclin, docetaxel	Gastrojejunostomy, colostomy	1.5
7	Puglisi et al. [18]	Anus	LN, peritoneum	Rectum	Anastrozole	No	3
8	Vennapusa et al. [19]	Stomach	LN, bone, orbit	No	Chemo, anastrozole	No	NM
9	López Deogracias et al. [20]	Rectum	Bone	No	Chemo	No	<1
10	Amin et al. [21]	Rectum	Axillary LN	No	AI	Hartmann	NM
11	Okido et al. [22]	Transverse	No	No	Trastuzumab	Segmental colectomy	≥1
12	Koike et al. [23]	Stomach	No	No	Toremifen, CMF, vinorelbine+ trastuzumab	No	≥2
13	Koike et al. [23]	Stomach	Bone, liver, peritoneum	No	AC, paclitaxel, letrozole, capecitabine, CMF	No	5
14	Saied et al. [24]	Small bowel	No	No	Fulvestrant	R colectomy	NM
15	Zhao et al. [25]	Duodenum	Brain	Cerebral	Chemo	Percutaneous biliary drain	NM
16	Critchley et al. [26]	Stomach, R colon	No	No	Docetaxel, anastrozole, capecitabine, epirubicin	No	NM
17	Saranovic et al. [27]	Rectum	Peritoneum	No	AI	Colostomy	≥1
18	Arrangoiz et al. [28]	Rectum, stomach	Axillary LN, liver, lung, bone	No	Paclitaxel, bevacizumab	No	≥1
19	Carcoforo et al. [29]	Sigmoid	Peritoneum	No	Doxorubicin, letrozole	Colectomy + colostomy	<1
20	Zuhair and Maron [30]	Stomach	No	No	Chemo, hormonal	No	3
21	Eren et al. [31]	Stomach	Peritoneum, ovary	No	Eribulin	No	<1
22	Kayılıoğlu et al. [32]	Stomach	Axillary LN	NM	NM	No	NM
23	Jones et al. [33]	Ampulla of Vater, duodenum	Axillary LN, liver	NM	NM	Colostomy	NM
24	Molina-Barea et al. [34]	Colon	Peritoneum	No	AI, paclitaxel	Exploratory laparotomy	1
25	Geredeli et al. [35]	Stomach	Bone	No	FAC, paclitaxel-carboplatin	Partial gastrectomy	NM
26	Dória et al. [36]	Stomach	Liver, peritoneum	No	Letrozole	No	NM
27	Buka et al. [37]	Stomach, colon, rectum	No	RT (stomach) + chemo	Capecitabine, paclitaxel, letrozole	Total gastrectomy	7
28	Balakrishnan et al. [38]	R colon, transverse	Liver, bone	NM	Fulvestrant+ everolimus	No	NM
29	Villa Guzman et al. [39]	Stomach, colon	Bone, BM, brain	No	Capecitabine, carboplatin-paclitaxel-BEV, cisplatin-gemcitabin, VNB, lipos. doxorubicin	No	2
30	Lau et al. [40]	Rectum	No	Rectum	Hormonal	Colostomy	2
31	Khokhlova et al. [41]	Jejunum	No	No	No	Intestinal resection + enteroanastomosis	NM
32	Cherian et al. [42]	Rectum	No	No	Letrozole	No	<1
33	Horimoto et al. [43]	Stomach, transverse, rectum	Bone	No	AI, capecitabine	No	≥1
34	Xu et al. [44]	Stomach	Bone, lung, pleura, skin	No	Letrozole	No	≥1
35	Koufopoulos et al. [45]	Sigmoid	No	No	NM	Low anterior resection	NM
36	Invento et al. [46]	Duodenum	Other breast	No	NM	No	NM
37	Asmar et al. [47]	Stomach	No	No	Fulvestrant	No	NM
38	Ogawa et al. [48]	Stomach	Brain	No	No	Stent	0.5
39	Falco et al. [49]	Colon	Other breast	No	NM	R colectomy	NM
40	Ruymbeke et al. [50]	Anus	Bone	Yes	Everolimus, EC	Colostomy	1.2
41	Woo et al. [51]	Stomach	Bone, ovary, peritoneum, BM	No	Yes	Gastrectomy	5.2
42	Martins Figueiredo et al. [52]	Colon	Orbit, LN	No	Yes	No	NM
43	Mosiun et al. [53]	Colon	Bone, ovary, breast, peritoneum	No	Paclitaxel	R colectomy	NM
44	Sharbatji et al. [54]	Terminal ileum, R colon	No	No	NM	R colectomy	NM
45	Numan et al. [55]	Small bowel, appendix	Breast, lung, ovaries	No	NM	Ileocecectomy, appendectomy, lysis of adhesions	NM
46	Blachman-Braun et al. [56]	Colon	No	No	No	No	NM
47	Gangireddy et al. [57]	Small bowel	No	No	Hormonal	Small bowel resection	NM
48	Noor et al. [58]	Stomach, sigmoid	Bone	No	Hormonal, chemo	No	7
49	Sarfraz et al. [59]	Rectosigmoid	Peritoneum	No	Hormonal, chemo	No	NM
50	Meekel et al. [60]	Terminal ileum, cecum	Peritoneum	No	No	Colectomy	<1
51	Kaneko et al. [61]	Stomach	No	No	Hormonal, chemo	No	NM
52	Kobayashi et al. [62]	Stomach, colon	Bone	No	Hormonal	No	1,2
53	Fu et al. [63]	Stomach	Bone	Yes	Hormonal, chemo	No	NM
54	Kutasovic et al. [64]	Stomach	No	No	Chemo	Subtotal gastrectomy	1
55	Fontes et al. [65]	Rectum	No	No	No	Derivative colostomy	<1
56	Alfian Sulai et al. [66]	Esophagus	Bone	No	Hormonal, chemo	No	NM
57	Abdallah et al. [67]	Transverse	Bone	No	Hormonal, chemo	No	NM
58	Abdallah et al. [67]	Stomach, small bowel	Peritoneum, bone, ovaries	No	Chemo	No	NM
59	Maharajh et al. [68]	Small bowel-terminal ileum	No	NM	NM	Ileocecectomy, partial colectomy	NM
60	Ali et al. [69]	Stomach	Bone	No	Hormonal	No	>1
61	Almahmeed et al. [70]	Rectum	Bone	No	Hormonal, chemo	Laparoscopic diverting loop ileostomy	NM
62	Zhang et al. [71]	Stomach	No	No	Hormonal, chemo	No	NM
63	Algethami et al. [72]	Sigmoid	NM	NM	NM	NM	NM
64	Mazza et al. [73]	Rectum	No	No	Chemo	No	NM
65	Zengel et al. [74]	Stomach	No	No	Hormonal	No	<1
66	Zengel et al. [74]	Transverse	Bone	No	Hormonal	No	NM
67	Imai et al. [75]	Colon	Bone	No	Hormonal	No	NM
68	Kimchy et al. [76]	Stomach, colon	Bone, liver	No	No	No	<1
69	Katuwal et al. [77]	Stomach	No	No	Hormonal, chemo	No	>2
70	Sun et al. [78]	Stomach	Bone	No	Hormonal, chemo	No	NM
71	Martino et al. [79]	Colon	Bone	No	Hormonal, chemo	No	3
72	Shin et al. [80]	Stomach	No	No	Hormonal, chemo	No	NM
73	Anguiano-Albarran et al. [81]	Stomach	No	No	No	No	<1
74	Ito et al. [82]	Stomach	Bone	No	Hormonal, chemo	No	>5
75	Ito et al. [82]	Stomach	No	No	Hormonal, chemo	No	NM
76	Yoshida et al. [83]	Colon	Bone	No	No	No	0.5
77	Li et al. [84]	Ileum, colon, rectum	No	No	Hormonal	No	0.6
78	Li et al. [85]	Stomach	Ovaries, peritoneum	No	NM	No	NM
79	Li et al. [86]	Small bowel	Breast	No	Chemo, hormonal	Small bowel resection	NM
80	Barbieri et al. [87]	Duodenum, stomach	No	No	Hormonal	Whipple	2
81	Kachi et al. [88]	Sigmoid, appendix	Ovaries	No	NM	Sigmoidectomy	NM
82	Namikawa et al. [89]	Stomach	Bone	No	Hormonal	No	0.75
83	Yanagisawa et al. [90]	Transverse	No	No	Hormonal	R colectomy	0.2
84	Skafida et al. [91]	Stomach, colon	Skin	No	Chemo, hormonal	No	2
85	Hamidu et al. [92]	Right and transverse colon	No	Yes	Hormonal, chemo	Yes	>2
86-112	McLemore et al. [93]	NM	NM	NM	NM	NM	2.5
113	Ayantunde et al. [94]	Esophagus	LN	No	NM	Dilatation + esophageal stent	1
114	Ayantunde et al. [94]	Cardia	Bone, peritoneum	No	Hormonal	Stent	8
115	Ayantunde et al. [94]	Cardia	Bone, liver	No	Hormonal	No	1.5
116	Ayantunde et al. [94]	Stomach	Bone, peritoneum, LN	No	No (BSC)	No	2
117	Ayantunde et al. [94]	Stomach	Bone, peritoneum, brain	No	No (BSC)	No	<1
118	Ayantunde et al. [94]	Stomach	Bone	No	Chemo	Gastrojejunostomy	1
119	Ayantunde et al. [94]	Stomach	Bone, peritoneum, LN	No	Hormonal	Stent	<1
120	Pectasides et al. [95]	Stomach	Bone, ovary	NM	FAC	NM	1
121	Pectasides et al. [95]	Stomach	No	NM	CMF, TAM	NM	<1
122	Pectasides et al. [95]	Stomach	No	NM	Xeliri+letrozole	NM	4
123	Pectasides et al. [95]	Stomach	Bone, peritoneum	NM	Epirubicin, paclitaxel	NM	<1
124	Pectasides et al. [95]	Stomach	Bone, peritoneum	NM	CMF, TAM, letrozole	NM	<1
125	Pectasides et al. [95]	Stomach	No	NM	Epirubicin, docetaxel	NM	3
126-132	Mathew et al. [96]	NM	NM	NM	NM	NM	NM
133-162	Montagna et al. [97]	NM	NM	NM	NM	NM	NM
163	Hong et al. [98]	Stomach	Meninges	No	Hormonal, chemo	No	NM
164	Hong et al. [98]	Stomach	No	No	Hormonal, chemo	No	13.3
165	Hong et al. [98]	Stomach	Bone	No	Chemo	No	8
166	Hong et al. [98]	Stomach	Liver	No	Chemo	No	7.3
167	Hong et al. [98]	Stomach	No	No	Hormonal, chemo	No	4.3
168	Hong et al. [98]	Stomach	No	No	Chemo	No	3.5
169	Hong et al. [98]	Stomach	Bone, ovary, brain	No	Hormonal, chemo	No	10.4

At least one systemic treatment was given to most of the patients for GI metastatic disease. Among 93 patients with available information on treatment, 83 (89.2%) received systemic therapies for GI metastases, of which 35 patients (37.6%) were treated with successive therapies (hormonal, chemotherapy), 25 (26.9%) with hormonal therapy only, 19 (20.4%) with chemotherapy only, and the rest with other options. Ten patients (10.8%) underwent only surgery, without any systemic treatment.

Concerning hormonal treatment for GI metastases, aromatase inhibitors (letrozole, anastrozole) were mostly used, whereas selective estrogen receptor modulators (tamoxifen and toremifene) were also administered to some patients. In addition, fulvestrant alone or in combination with everolimus was also given to three patients [24,38,47]. In the most recent reports, cyclin-dependent kinase 4/6 (CDK4/6) inhibitors, combined with AI or fulvestrant, were frequently chosen. Given that the hormonal agents were not always reported, we did not calculate the exact frequency of each drug.

Most common chemotherapy regimens included taxanes and anthracyclines, in line with advanced breast cancer guidelines. Other drugs were also used, such as capecitabine, carboplatin, vinorelbine, and eribulin. Two patients received the monoclonal antibody bevacizumab in combination with chemotherapy [28,39]. Two patients received anti-HER2 treatment (trastuzumab) without concomitant chemotherapy [22,23].

Only four patients were treated with radiation therapy delivered to GI metastases, two of which to the rectum [18,40], one to the anus [50] and one to the stomach [37]. The latter was considered initially as a primary gastric tumor, and concomitant radiotherapy with chemotherapy was chosen.

Finally, 40 patients (40.4%) underwent a kind of intervention, more or less invasive, for their GI metastasis, 59 patients (59.6%) did not, whereas for the remaining 71 patients the information was not available (Table 3). Several patients underwent emergent surgery for intestinal obstruction, including small bowel resection (n=5) and colostomy (n=8). More sophisticated surgeries were performed on patients with gastrointestinal tumors initially considered primary, including the Whipple procedure (n=2), gastrectomy (n=5), colectomy (n=9), and abdominoperineal resection (n=1). Another four patients required an esophageal stent. 

Survival data was not available for the whole population; therefore, we analyzed only a limited number of cases. Median survival since diagnosis of GI metastasis is seven years (one month to 13.3 years, 95% CI: 2.9-11.1). We did not perform any subgroup analysis to identify potential associations of survival with metastatic sites or different treatments, given the small sample size.

Discussion

This review represents an analysis of the 169 reported cases of gastrointestinal metastases from lobular breast carcinoma, in terms of histological characteristics of both primary tumors and metastases, different treatments, and outcomes. The majority of cases were HR-positive, HER2-negative luminal carcinoma, as expected for luminal breast carcinoma. Our analysis was limited to lobular carcinoma, as they are more likely to metastasize to the GI tract [99]. A large retrospective series of 4140 patients demonstrated that IDC has a probability of 1.1% of GI metastasis, versus 4.5% in ILC [4].

One possible explanation is the loss of E-cadherin expression in ILC. Epithelial cadherin or E-cadherin belongs to a class of trans-membranous proteins and is critical in tumor progression, functioning as suppressor of invasion and metastasis in numerous contexts. They play a crucial role in cell-to-cell contact formation and stability, as they mediate cell-cell adhesion within tissues. Their function depends on calcium ions, which is the root of their names [100].

It should be noted that most patients with metachronous GI metastases were initially diagnosed with stage III disease. However, four patients presented with metastases eight to 10 years after stage I breast carcinoma diagnosis [14,40,41,63].

Metastases of the GI tract are rare in general. Breast cancer and in particular ILC is one of the most common primary tumors able to metastasize to this location. Furthermore, melanoma, lung cancer, renal cancer, ovarian cancer, and pancreatic cancer have also been associated with secondary GI localizations [101,102].

In the case of GI lesion, an initial diagnosis of primary cancer of the GI tract is evocated, especially when the time interval from breast cancer diagnosis is long. Histological examination should point towards a metastatic lesion but correct diagnosis can be challenging in some circumstances. One such case relates to signet ring morphology in microscope, where the lesion can be misdiagnosed as primary gastric carcinoma [23,37,43-45,95]. Immunohistochemistry is very important in these cases, namely ER and PR antibodies. Another challenging situation is where HR is negative, as it was shown in some of the GI metastases described. The majority of cases of gastric metastases were initially diagnosed as primary gastric tumors (given mainly the presence of signet ring cells).

Upper GI and more precisely stomach was the most common metastatic site. Our analysis confirms previous findings that gastric metastases are associated with the worst prognosis, with survival of no more than 1-1.5 years in most cases [43,95], with only a few exceptions [18,30,37,58,79,94,98]. The prognosis of the whole population is in general poor, compared to patients with other metastases such as bone. It should be noted, however, that the follow-up time was generally short and the sample size relatively small, which makes it difficult to draw reliable conclusions about survival.

ILC patients should be treated similarly to IDC patients. Patients with HR-positive and HER2-negative disease should be treated with endocrine therapy. Currently, CDK4/6 inhibitors combined with non-steroidal aromatase inhibitors showed progression-free survival (PFS) benefits against aromatase inhibitor monotherapy and constitute the preferred regimen in the first-line setting. The results of PALOMA-2, MONALEESA-2, and MONARCH-3 trials led to FDA and EMA approval of palbociclib, ribociclib, and abemaciclib respectively [103-105]. PALOMA-2 trial, in particular, showed significantly improved PFS (HR: 0.58; 95% CI: 0.46-0.72; p < 0.001) in both ILC patients (HR: 0.46) and patients who had visceral metastasis (HR: 0.63), whereas no data about ILC have been reported for ribociclib and abemaciclib [105,106]. In addition, prolonged PFS and overall survival (OS) were reported with CDK 4/6 inhibitors in combination with fulvestrant versus fulvestrant monotherapy in the second-line setting [107-109]. The three available drugs have different adverse events, with neutropenia being the most common dose-limiting toxicity of both ribociclib and palbociclib and diarrhea of abemaciclib. The toxicity profile of each CDK4/6 inhibitor should be considered, especially in patients with GI metastases who have probably already GI symptoms.

Given that patients with gastrointestinal metastases from lobular breast carcinoma should be treated similarly to patients with metastatic breast cancer, early detection of these cases is crucial. Our findings highlight that clinicians (both surgeons and medical oncologists) should be aware of the possibility of GI metastases and obtain a complete and accurate medical history in case of GI tumors, before any treatment decision. Furthermore, in case of medical history of ILC, clinicians should alert pathologists examining GI tumors, as differential diagnosis from primary GI cancers is sometimes challenging. 

Our study has certain limitations, primarily its retrospective nature, which is associated with a potential selection bias. In addition, the variability in treatment regimens and treatment modalities does not allow a proper assessment of the optimal treatment. The role of local therapies (surgery, radiation therapy) cannot be further explored in this analysis.

## Conclusions

We analyzed the clinical presentation and outcomes of 170 patients with GI metastases from lobular breast carcinoma, most of which had HR-positive, HER2-negative tumors. We conclude that GI metastases are rare and can arise in every part of the GI tract, with the stomach being the common site. Usually, they are initially considered as primary tumors of the GI tract, even if there is a history of breast cancer. A careful histological examination, including specific immunohistochemical biomarkers for breast carcinoma, is the key to the right diagnosis. GI metastases from lobular breast carcinoma should be included in the differential diagnosis of GI lesions and should be managed according to advanced breast cancer algorithms.
